# Visible-Light-Driven AO7 Photocatalytic Degradation and Toxicity Removal at Bi-Doped SrTiO_3_

**DOI:** 10.3390/ma15072465

**Published:** 2022-03-27

**Authors:** Maria João Nunes, Ana Lopes, Maria José Pacheco, Lurdes Ciríaco

**Affiliations:** Fiber Materials and Environmental Technologies (FibEnTech-UBI), Universidade da Beira Interior, R. Marquês de D’Ávila e Bolama, 6200-001 Covilhã, Portugal; analopes@ubi.pt (A.L.); mjap@ubi.pt (M.J.P.); lciriaco@ubi.pt (L.C.)

**Keywords:** perovskite, Bi-doped SrTiO_3_, visible light photocatalysis, AO7, toxicity

## Abstract

In this study, Bi-doped SrTiO_3_ perovskites (Sr_1−x_Bi_x_TiO_3_, x = 0, 0.03, 0.05, 0.07 and 0.1) were synthesized using the solid-state method, characterized, and tested as photocatalysts in the degradation of the azo dye acid orange 7 (AO7) under visible light. The perovskites were successfully synthesized, and XRD data showed a predominant, well-crystallized phase, belonging to the cubic perovskite symmetry. For the doped samples, a minority phase, identified as bismuth titanate, was detected. All doped samples exhibited improved photocatalytic activity under visible light, on the degradation of AO7 (10 mg L^−1^), when compared with the undoped SrTiO_3_, with an increase in relative Abs_484 nm_ decay from 3.7% to ≥67.8% after 1 h, for a powder suspension of 0.2 g L^−1^. The best photocatalytic activity was exhibited by the Sr_0.95_Bi_0.05_TiO_3_ perovskite. Reusability studies showed no significant loss in photocatalytic activity under visible light. The final solutions showed no toxicity towards *D. magna*, proving the efficiency of Sr_0.95_Bi_0.05_TiO_3_ as a visible-light-driven photocatalyst to degrade both the AO7 dye as well as its toxic by-products. A degradation mechanism is proposed.

## 1. Introduction

Photocatalysis is a promising, clean process that can be applied in the degradation of persistent organic pollutants, such as organic dyes [1,2,3,4,5,6]. This process initiates with the irradiation of a suitable catalyst’s surface with a type of radiation, i.e., UV, visible, or solar, with energy equal to or higher than the bang gap energy (E_g_) value. If this prerequisite is met, the electrons of the valence band are excited to the conduction band, initiating a succession of reactions that culminate with the formation of highly reactive radical species, such as O^2•−^ and OH^•^. These radical species will react with the organic molecules, breaking the bonds and generating intermediate metabolites and leading to, ideally, complete mineralization.

A photocatalyst can exhibit activity under visible light if its E_g_ is narrow enough to be activated by visible radiation [7]. This facilitates the application of natural sunlight as the source of renewable energy, given that ~42% of the incident radiation falls under the visible wavelength, resulting in a more cost-effective and environmentally friendly process. Furthermore, the reuse of treated effluents can be implemented according to the European Union (EU) regulation 2020/741 “on minimum requirements for water reuse”, for irrigation purposes and groundwater recharge [8].

Perovskite-type compounds, such as halide perovskites, have been studied as promising photocatalysts for some applications such as CO_2_ reduction under UV–visible radiation [9] and PET–RAFT polymerization under visible and near-infrared radiation [10], due to their superior properties. Other types, such as perovskite oxides, have shown to be more suitable than their halide counterparts for application as catalysts in the photocatalytic degradation of aqueous solutions of several compounds, including organic dyes, due to their higher water stability [11,12,13,14,15,16,17,18,19,20,21,22,23]. These ceramic materials, with the general formula ABO_3_, are well known for their stable and flexible structure that allows the accommodation of several cationic combinations and consequent variety of properties. Strontium titanate (SrTiO_3_) is a cubic perovskite oxide reported to exhibit photocatalytic activity under UV light [24,25]. This limitation is attributed to a wide energy gap of 3.0–3.2 eV [26,27,28] and hinders the application of low-cost solar light. One method to lower the energy gap value and increase the photoabsorption range of SrTiO_3_ is element doping, due to its low cost and overall simplicity [29]. Hou et al. [30] and Liu et al. [31] reported that through the Cr-doping of SrTiO_3_, E_g_ of 2.32–2.12 eV and lower than 3.2 eV were obtained, respectively. Comparable lowering of E_g_ of SrTiO_3_ was also observed by doping it with Mn (2.76 eV) [32] and Bi (2.6 eV and 3.04 eV) [33,34].

Azo dyes are a widely used group of synthetic dyes, accounting for 60–70% of the global industry demand [35,36]. These dyes are characterized by the presence of the -N=N- chromophore in diverse complex aromatic structures and possess high chemical stability and breakdown resistance over time to sunlight radiation and microorganisms [37]. Due to these characteristics, conventional wastewater treatments are not always effective to achieve complete degradation of these compounds. Azo dyes’ incomplete degradation could lead to the formation, and consequent release, of metabolites with higher toxicity than the parent dye molecule [36]. In the case of acid orange 7 dye, some of the degradation products are known to be more toxic than the parent compound, such as 1-amino-2-naphthol [38,39], 1,4-benzoquinone, and 2-naphthol [40]. 

In a previous study [41], our group studied Ni/Bi-doped SrTiO_3_ perovskite films on the degradation of AO7 under visible light, with the best results obtained by the Ni/Sr_0.9_Bi_0.1_TiO_3_ film, in which a relative Abs_484 nm_ decay of 83% was achieved after 7 h. These results showed to be promising but restricted to the immobilization technique applied and consequent reduction in surface area. Additionally, studies regarding the influence of experimental parameters on the photocatalytic activity, reusability, and degradation mechanism, were necessary to understand the catalytic behavior of the Bi-doped SrTiO_3_ perovskites.

In this study, Bi-doped SrTiO_3_ perovskites (Sr_1−x_Bi_x_TiO_3_, x = 0, 0.03, 0.05, 0.07 and 0.1) were synthesized, characterized, and tested as photocatalysts in the degradation of the AO7 dye under visible light. The influence of the amount of doped Bi in the perovskite structure and photocatalytic activity was studied. For the oxide with the best photocatalytic activity, the influence of initial pH, catalyst dose, initial dye concentration, and reusability was assessed, and a possible mechanism is discussed. Acute toxicity assays with *D. magna* were performed to the final solutions. To the best of our knowledge, the influence of Bi-doping of SrTiO_3_ on the photocatalytic degradation of organic molecules, under visible light, and on toxicity removal has not been reported.

## 2. Materials and Methods

### 2.1. Perovskite Powders Preparation and Characterization

The Sr_1−x_Bi_x_TiO_3_ (x = 0, 0.03, 0.05, 0.07 and 0.1) perovskite powders were prepared by the solid-state method, in which stoichiometric amounts of SrCO_3_ (Merk Life Science S.L., Algés, Portugal, ≥99.9%), Bi_2_O_3_ (Sigma-Aldrich, Algés, Portugal, ≥99.9%), and TiO_2_ (Sigma-Aldrich, Algés, Portugal, ≥99.5%) were ground in an agate mortar and heated (tubular furnace Carbolite, model STF, with a Carbonite Gero Type 3216 temperature controller (Sheffield, UK)), at 900 °C (x = 0) or 750 °C (0.03 ≤ x ≤ 0.1) for 24 h. The obtained samples were then reground and reheated at 1200 °C (x = 0) or 750 °C (0.03 ≤ x ≤ 0.1) for 24 h.

X-ray diffractograms (XRD) for all intermediate and final samples were obtained between 2θ = 10 and 90° at a scanning rate of 1.2°/min (Rigaku diffractometer, model DMAX III/C, plus APD Philips v3.5B (Philips, Lisbon, Portugal)) equipped with a monochromatized Cu kα radiation (λ = 0.15406 nm), operating at 40 mA and 30 kW). Results were handled in the Unitcell refinement program [42].

The powder samples’ morphological characterization was determined by scanning electron microscopy (SEM) (Hitachi, Tokyo, Japan, model S2700, at 20 keV).

Diffuse reflectance spectra (DRS) were measured with a SPEC STD spectrometer (Sarspec), configured with a 25 µm slit, light source, reflectance probe, and reflectance standard, operating in the ultraviolet–visible range using a Deuterium Tungsten High Power. The bandgap energies were calculated from the diffuse reflectance values through the Kubelka–Munk function (Equation (1)), where *R* is the diffuse reflectance of the samples, and *K* and *S* are the absorption and scattering coefficients, respectively.

(1) $$F\left(R\right)=\frac{{\left(1-R\right)}^{2}}{2R}=\frac{K}{S}$$

The E_g_ for all samples were determined by extrapolating the linear region of the Tauc plot, with (*F*(*R*).hc/λ)^2^ vs. hc/λ, to the abscissa axis, where h is the Planck’s constant, c is the speed of light, and λ is the wavelength at the corresponding reflectance. 

### 2.2. Photocatalytic Activity under Visible Light

For the photocatalytic experiments with Sr_1−x_Bi_x_TiO_3_ (x = 0, 0.03, 0.05, 0.07 and 0.1) samples, 0.002 g or 0.005 g of each powder were dispersed in 10 mL of a 10 mg L^−1^ AO7 (Sigma-Aldrich, Algés, Portugal, 85%) aqueous solution. All suspensions were sonicated for 15 min and stirred in the dark for 45 min, to achieve the adsorption–desorption equilibrium between the dye molecules and the catalyst, after which they were irradiated by visible light (Osram, 300 W (Phillips, Lisbon, Portugal), emission spectrum in Supplementary Materials, Figure S1) for 1 h. For the assays performed with the Sr_0.95_Bi_0.05_TiO_3_, an amount of the powders (0.01, 0.02, and 0.05 g) was dispersed in 100 mL of an aqueous solution of AO7 (10, 25, and 50 mg L^−1^). The same procedure was followed to achieve the adsorption–desorption equilibrium. The suspensions were irradiated by the same light source for 2 h. For some assays, the pH was adjusted with aqueous solutions of HCl or NaOH. At given intervals, samples of 3 mL were collected and centrifuged, at 5000 rpm for 5 min, and the supernatant was collected. The dye degradation was monitored by UV–visible absorption spectrophotometry (Shimadzu UV-1800 spectrophotometer, Tokyo, Japan), between 200 and 600 nm.

For the optimal experimental conditions, the dye concentration was monitored by reverse-phase high-performance liquid chromatography (RP-HPLC, Shimadzu, Tokyo, Japan), as well as the formation of degradation products, such as sulfanilic acid (SA; Panreac, Applichem, Prior Velho, Portugal, 99.5%), 1-amino-2-naphthol (AN; Sigma-Aldrich, Algés, Portugal, 90%), 1,4-benzoquinone (BQ; Acros Organics, Fisher Scientific, Lisbon, Portugal, 99%), and hydroquinone (HQ; Fluka Analytical, Sigma-Aldrich, Algés, Portugal, ≥99.0%). HPLC analysis was performed in a Shimadzu 20A Prominence HPLC system (DAD-SSPD-M20A; Merck Millipore column, with Purosphere STAR RP-18 endcapped (250 mm × 4 mm (i.d.) and 5 µm particles)). The chromatographic conditions were adapted from previous research [25]. A mixture of 33 mM phosphate buffer (pH 7.0) (KH_2_PO_4_, Fisher Scientific, Lisbon, Portugal, HPLC grade; K_2_HPO_4_, AnalaR, 99%) (component A) and acetonitrile (Fisher Chemical, Algés, Portugal, ≥99.99%, HPLC grade) (component B) was used as the eluent, and the elution was performed at 0.7 mL min^−1^, with a relative percentage of B of 20% for 9 min, increasing to 40% until 11 min, and maintained until 25 min. Detection was carried out at a wavelength of 249 nm for SA, 252 nm for AN, 254 nm for BQ, 288 nm for HQ, and 484 nm for AO7. Carboxylic acids of low molecular weight were detected by ion exclusion chromatography, using the same HPLC apparatus and a Biorad Aminex HPX-87H column (300 mm × 7.8 mm (i.d.)) at 35 °C and at a wavelength of 210 nm for all acids. A 4 mM sulfuric acid aqueous solution was used as eluent, and the elution was performed isocratically at 0.6 mL min^−1^. All mobile phases and samples were filtered with 0.45 µm and 0.22 µm filters (Whatman, Maidstone, UK), respectively, and the injection volume was 20 µL for both methods.

Acute toxicity assays with *D. magna* were performed using the commercial Daphtoxkit F^TM^ Magna Test Kit, following the OECD/OCDE Guideline 202 [43]. The used batch, DM090419, exhibited a mean 24 h EC_50_ for the reference toxicant potassium dichromate of 1.26 mg L^−1^, which is within the acceptability range of 24 h EC_50_ 0.6–2.1 mg L^−1^ [43]. Standard freshwater, reproducing natural freshwater, was prepared and used in the hatching of the ephippia, prior to toxicity tests, and in the dilution sets for each solution tested. After 2 h and 6 h, solutions resulting from assays that were run under optimal experimental conditions were centrifuged, and the supernatant was filtered with a 0.22 µm filter. From each of these solutions, a set of dilutions (5.625, 11.25, 22.5, 45, and 90%) were prepared with the standard freshwater and poured into four 10 mL wells in the test plate. In each well, 5 neonates (with less than 24 h) were transferred and incubated in the dark for 48 h, at 20 °C. All neonates unable to swim for 15 s, after gentle agitation of the solution, were considered immobilized and registered after 48 h, and the EC_50_ was calculated.

## 3. Results and Discussion

### 3.1. Perovskite Powder Characterization

**Figure 1:**
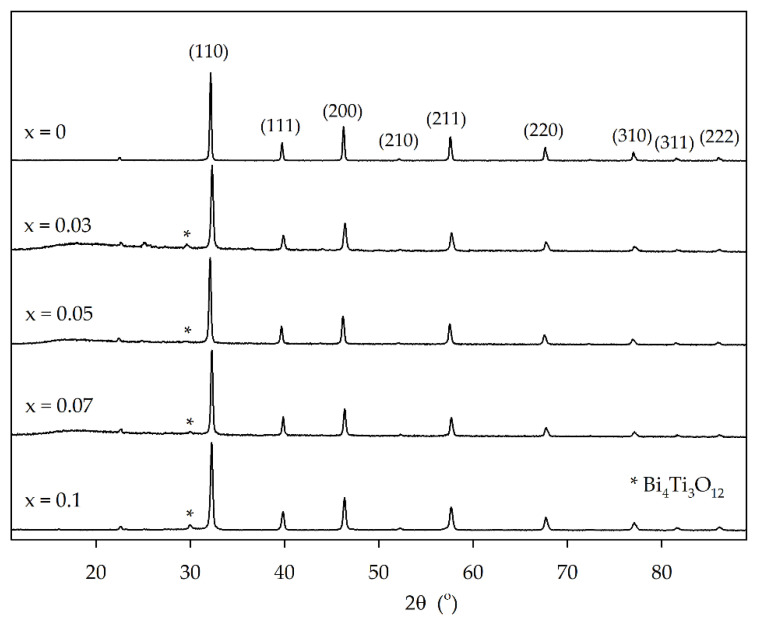
XRD patterns of the Sr_1−x_Bi_x_TiO_3_ powder samples.

Figure 1 presents the XRD patterns for the Sr_1−x_Bi_x_TiO_3_ (x = 0, 0.03, 0.05, 0.07 and 0.1) powder perovskites, in which it is possible to identify a predominant well-crystallized phase in all samples, belonging to the cubic perovskite symmetry in a *Pm*$\bar{3}$*m* space group (ICDD file PDF#35-0734). For Bi-doped samples, an extra minority phase was identified as belonging to bismuth titanate, Bi_4_Ti_3_O_12_ (ICDD file PDF#47-0398), by the presence of its most intense peak, at 29.9° [25].

The cell parameters and unit volume, presented in Table 1, vary slightly and in an irregular way with Bi doping, not obeying Végard’s law, which is in accordance with the obtained results, as a pure solid solution for the doped samples was not observed. With the substitution of Sr^2+^ cations, with ionic radii of 0.126 nm, by smaller Bi^3+^ cations, with 0.117 nm, the unit cell was expected to contract with the introduction of bismuth in the perovskite structure. However, the cell parameters did not follow this tendency and varied irregularly with Bi content. This corroborates the XRD results, showing that a pure solid solution was not obtained.

**Figure 2:**
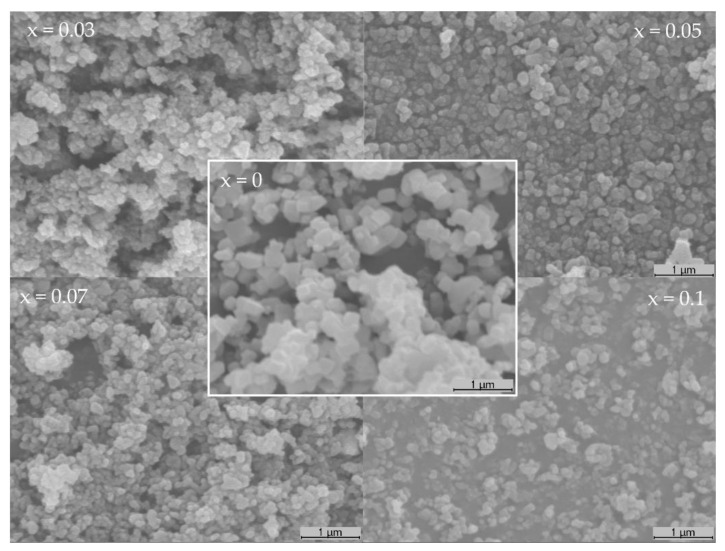
SEM micrographs of the Sr_1−x_Bi_x_TiO_3_ powder samples (×20,000).

The crystallite size (Table 1) was calculated by the Scherrer equation, using the full width at half maximum (FWHM) of the (110) peaks, and it is shown to have smaller values for the doped samples. Even though the crystalline size is known to be associated with the smallest particle size in powder form (most likely single crystal), a similar size variation was observed in the SEM images (Figure 2), with a significant reduction in the smaller observed particles when comparing the SrTiO_3_ to Bi-doped samples.

The average observed particle size was smaller for the Bi-doped samples than that for the undoped SrTiO_3_, with some exhibiting dimensions lower than 100 nm. Regarding the overall morphology, no significant difference was observed.

The calculated E_g_ values for all samples are presented in Table 1, which increased slightly for the doped samples, from 3.43 eV (SrTiO_3_) to 3.65–3.66 eV. These values indicate that all oxides are more suitable to be activated by UV light. However, given the identification of a secondary phase in the doped samples, and how it can provide a different layout of the conduction and valence bands [29,44,45], these oxides were tested under visible light.

### 3.2. Photocatalytic Activity under Visible Light

The AO7 dye was used as a model organic pollutant to evaluate the photocatalytic activity of the prepared Sr_1−x_Bi_x_TiO_3_ (x = 0, 0.03, 0.05, 0.07 and 0.1) perovskites. To analyze the dye content during the experiments, the absorbance values at 484 nm were monitored. The band at 484 nm belongs to the visible region of the UV–vis absorption spectrum and results from the conjugated system in the molecule [46]. Before studying the photocatalytic activity of the oxides, photolysis assays were performed to assess the contribution of the visible light radiation to the degradation of the dye. For an initial AO7 concentration of 10 mg L^−1^, a 1.0% Abs_484 nm_ decrease was observed after 1 h. After 2 h, for all tested AO7 initial concentrations, the relative Abs_484 nm_ decay was ≤5.8%. Adsorption assays were also performed (data not shown) for all tested oxides, to determine the time necessary to achieve the adsorption–desorption equilibrium between the dye and the catalyst. After 1 h, the amounts of adsorbed AO7 were ≤7.0% and ≤8.6%, for a catalyst dose of 0.2 and 0.5 g L^−1^, respectively.

**Figure 3:**
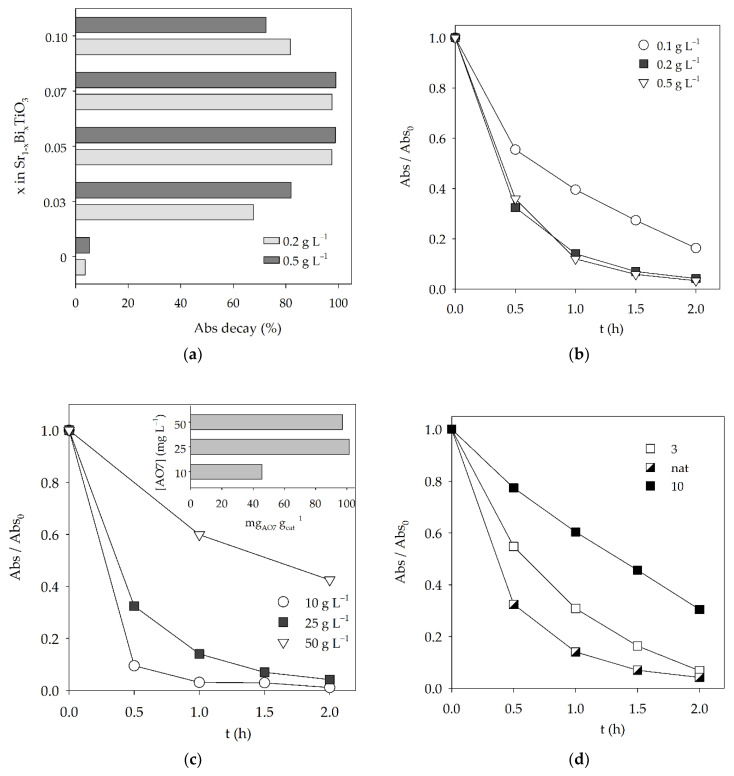
Influence of (**a**) Bi doping on the relative Abs_484 nm_ decay after 1 h (C_i_ AO7 = 10 mg L^−1^, at two catalyst doses); (**b**) catalyst dose; (**c**) initial dye concentration (inset mg AO7 removed/g of catalyst, for the tested dye concentrations); (**d**) pH on the relative absorbance decays, measured at 484 nm, for the photocatalytic degradation of AO7, with the Sr_0.95_Bi_0.05_TiO_3_ perovskite under visible light.

The effects of Bi doping on the photocatalytic activity of the perovskites under visible light were tested, and the results are presented in Figure 3a. The results show a poor relative absorbance decay for the strontium titanate sample, with 3.7% and 5.3% removals after 1 h irradiation for catalyst doses of 0.2 g L^−1^ and 0.5 g L^−1^, respectively. These values are in accordance with the calculated E_g_ value of 3.43 eV, which indicates that SrTiO_3_ would be more suitable to be activated by UV light. However, despite the similar high E_g_ values (3.65–3.66 eV), the Bi-doped samples exhibited a significant increase in the photocatalytic activity, when compared with the undoped SrTiO_3_, with relative Abs_484 nm_ decays above 97.5% after 1 h, for both Sr_0.95_Bi_0.05_TiO_3_ and Sr_0.93_Bi_0.07_TiO_3_ samples. This behavior could be due to the occurrence of the Bi_4_Ti_3_O_12_ secondary phase in all doped samples, alongside the Sr_1−x_Bi_x_TiO_3_ cubic perovskite phase, resulting in a synergetic effect between both phases, given that the Bi_4_Ti_3_O_12_ possess photocatalytic activity, achieving a relative Abs_484nm_ decrease of 27.6% after 2 h, for a catalyst dose of 0.2 g L^−1^, and under the same conditions (Supplementary Material Figure S2). Another possibility is the formation of a heterostructure, where the valence and conduction bands are distributed in such a manner that the recombination of photogenerated electron–hole pair could be reduced, increasing the photocatalytic activity of the samples with that structure [29,44,45]. However, given the available results, the latter cannot be confirmed. Further studies about the band structures and work function of the heterostructures need to be performed [47,48]. This effect in the catalytic activity for the degradation of AO7 (Figure 3a) increases significantly with the amount of doped bismuth until x = 0.1, where a decrease in the relative Abs_484nm_ decay is observed, indicating that there is an optimal ratio between both phases. The smaller particle size, observed for the doped samples (Figure 2), should also be considered, as it results in a higher surface area for the catalytic reaction to occur.

Given that the best results were obtained for the Sr_0.95_Bi_0.05_TiO_3_ perovskite, further studies were performed with this sample to infer the optimal experimental conditions for the AO7 photocatalytic degradation. Figure 3b shows the influence of the catalyst dosage in the degradation of 25 mg L^−1^ AO7 aqueous solutions. An increase in the initial catalyst dose from 0.1 g L^−1^ to 0.2 g L^−1^ resulted in an improvement in the decolorization rate throughout the 2 h assay, with observed Abs_484 nm_ decays increasing from 83.7% to 95.8% after 2 h. However, no improvement was observed with the increase in the powder concentration from 0.2 g L^−1^ to 0.5 g L^−1^. An initial increase in the catalyst concentration led to a higher decolorization rate, which could be associated with an increase in the available catalyst surface area, followed by no perceivable improvement for the highest tested concentration. This behavior could be due to the increase in the turbidity of the perovskite suspension, hindering the penetration of the visible light radiation and catalyst activation.

Regarding the influence of the initial dye concentration (Figure 3c), the relative Abs_484 nm_ decay decreased with the increase in the initial AO7 concentration from 98.9% to 57.5% after 2 h, for 10 mg L^−1^ to 50 mg L^−1^, respectively. When analyzing the amount of removed dye (mg of removed AO7 per gram of catalyst, Figure 3c inset), it is possible to observe that the value increased with the initial increase in dye concentration (from 45 to, approximately, 100 mg of AO7 removed per g of catalyst), followed by no significant variation for the highest dye concentration. This behavior could be explained by a higher concentration of AO7 molecules adsorbed at the available catalyst surface area for the 50 mg L^−1^ AO7 assay, delaying the degradation mechanism and leading to results similar to those obtained at a lower concentration of 25 mg L^−1^ [49].

It is known that the pH can influence the interaction between the dye molecules and the catalyst, as it can affect the catalyst surface and dye molecule charge, and subsequent AO7 adsorption [49]. As it can be seen in Figure 3d, the best results were obtained at the naturally occurring pH (~6) of the suspension, indicating that this pH value is ideal for the dye degradation mechanism to occur, as it could promote an ideal adsorption rate and subsequent photocatalytic reaction. It could also be related to the pH of the zero-point charge of the Sr_0.95_Bi_0.05_TiO_3_ catalyst.

**Figure 4:**
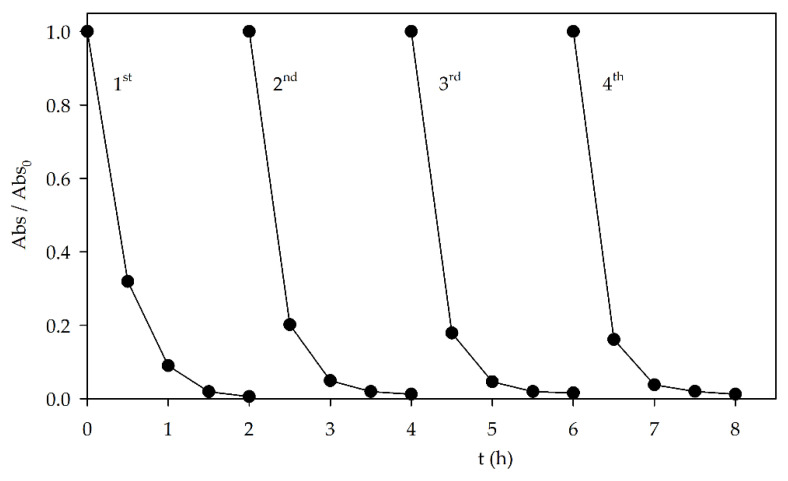
Reusability of the Sr_0.95_Bi_0.05_TiO_3_ catalyst (0.2 g L^−1^) on the photocatalytic degradation of AO7 (10 mg L^−1^) under visible light, during four cycles.

Reusability is an important parameter to take into consideration when testing a catalyst. To evaluate this characteristic, the perovskite was recovered after the photocatalytic experiment, washed with distilled water, and air-dried before being reused in the degradation of 10 mg L^−1^ AO7, using 0.2 g L^−1^ catalyst and without controlling the pH. The recovery percentage of the catalyst was between 78.2% and 80.2%. This percentage could be improved by using filters or membranes to facilitate the separation. However, this would increase the cost of the overall process. Results showed no significant loss in photocatalytic activity (Figure 4). Moreover, XRD and DRS analysis were performed on the used catalyst (Supplementary Material Figures S3–S5, respectively), with no significant alteration of the perovskite structure and cell parameters and calculated E_g_ values (3.65 eV). This shows that the Sr_0.95_Bi_0.05_TiO_3_ can be reused without significant loss of either photocatalytic activity under visible light or structural, morphological, and optical stability.

### 3.3. Analysis of the Degradation Products and Proposed Mechanism

**Figure 5:**
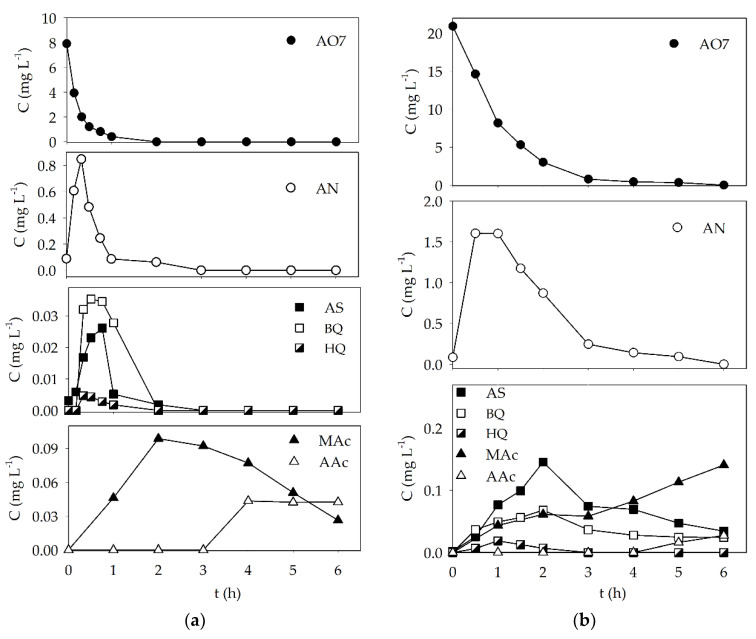
Concentration variation for the AO7 dye and some degradation products during the photocatalytic degradation of (**a**) 10 mg L^−1^ and (**b**) 25 mg L^−1^ of AO7 under visible light with Sr_0.95_Bi_0.05_TiO_3_ perovskite (0.2 g L^−1^).

Experiments carried out with Sr_0.95_Bi_0.05_TiO_3_ under optimal conditions and AO7 concentrations of 10 and 25 mg L^−1^ were performed for 6 h and monitored by RP-HPLC, to assess the dye concentration decay and detect some of the possible degradation by-products such as SA, AN, (previously detected and reported [25,50]) BQ, HQ, and some low-molecular-weight carboxylic acids (Figure 5).

The AO7 concentration decay was observed to occur primarily during the first and third hours of the experiments for initial dye concentrations of 10 mg L^−1^ and 25 mg L^−1^, respectively. SA and AN were detected during these times, with an increase in concentration until reaching a maximum value, followed by a decrease until the end of both assays. In the case of the experiment conducted with an initial dye concentration of 10 mg L^−1^, both metabolites were completely degraded after 3 h, which coincided with the formation and detection of some low-molecular-weight acids such as maleic and acetic acid (Figure 5). Formic acid was also detected but in very low concentration. Similar behavior was observed for the assays performed at 25 mg L^−1^, with the slower formation and degradation times for all detected metabolites. BQ and HQ were also detected under both experimental conditions, both presenting similar outline profiles as SA. 

This behavior shows that once the dye concentration is low enough, and diffusion is the main controlling step, the metabolites begin to suffer photocatalytic degradation as well, and in some instances, full degradation is achieved.

**Figure 6:**
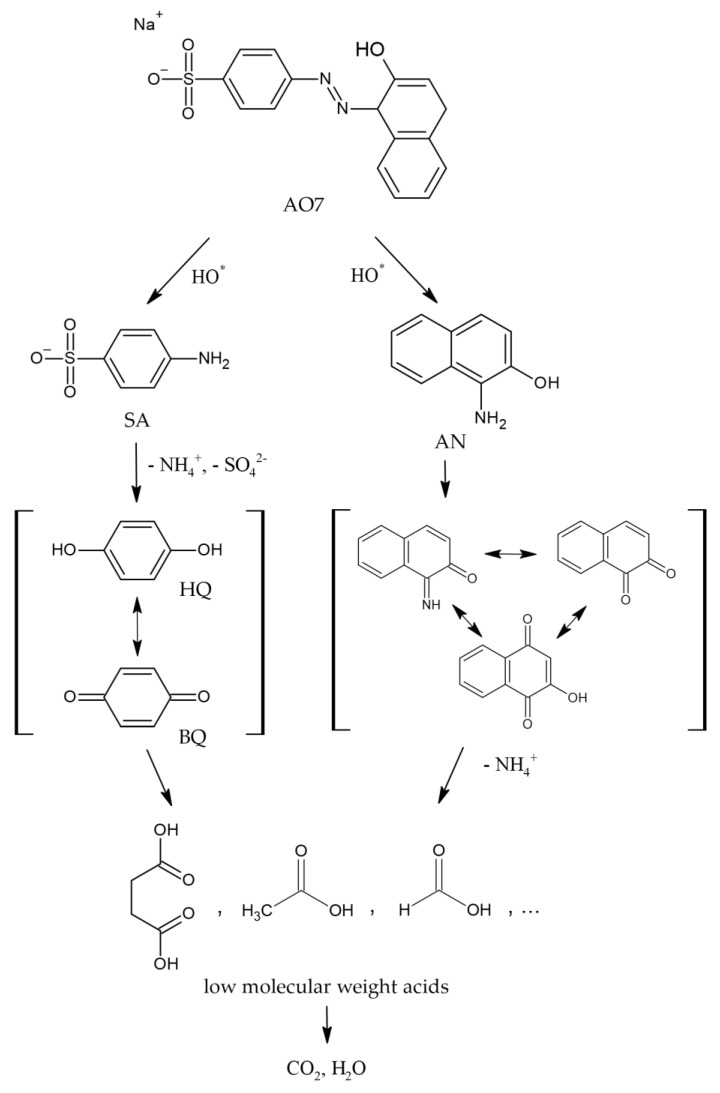
Proposed AO7 degradation pathway.

By analyzing the order of detection and overall outline of all monitored compounds, some insights were inferred, based on which a simple degradation mechanism is proposed in Figure 6.

In this proposed mechanism, it was assumed that the cleavage of the azo bond is the most likely first step in the degradation pathway [51], which is corroborated by the detection of the main reaction products AN and SA, formed shortly after the degradation assays started and AO7 concentration began to decrease. SA could suffer further oxidation into other detected benzenic compounds such as HQ and BQ, which, as previously mentioned, had similar outlines as SA. The same could occur with AN, which, initially, led to the formation of other naphthalenic compounds. The formation of low-molecular-weight acids follows the opening of benzenic and naphtalenic rings and further oxidation reactions, and it occurs after a significant concentration of the preceding high-molecular-weight metabolites is already decreasing. Ideally, the degradation pathway leads to the total mineralization of the molecules into CO_2_, H_2_O, and other inorganic substances.

TOC values were determined for both assays, after 2 and 6 h, with results presented in Table 2. Good mineralization was observed after 6 h for the assay with 10 mg L^−1^, with 42.3% TOC removal, which could indicate that with enough time, almost complete mineralization could be achieved. For the assay at 25 mg L^−1^, the low TOC removals (14.4% and 19.4% after 2 h and 6 h, respectively) are in accordance with the detection of several metabolites at both times.

### 3.4. Kinetics

**Figure 7:**
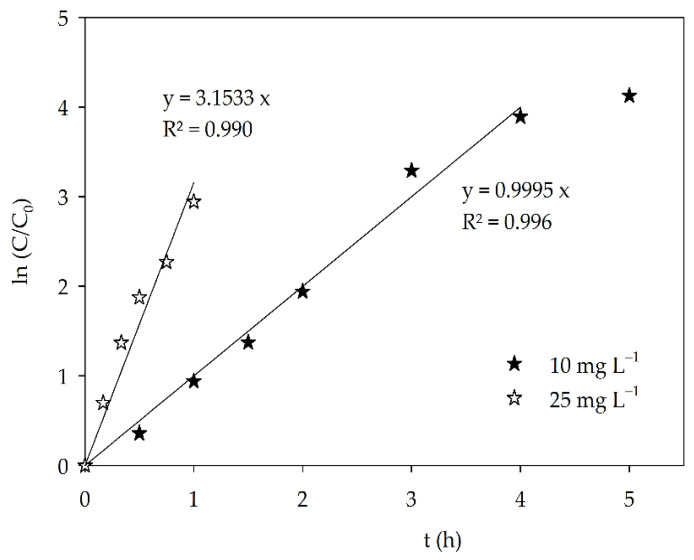
Pseudo-first-order kinetics for AO7 degradation under visible light over Sr_0.95_Bi_0.05_TiO_3_.

As it can be seen in Figure 5, the dye concentration decreased exponentially with the irradiation time, meaning that it can be described by a pseudo-first-order rate model. The apparent rate constant (k) can be calculated as ln(C_0_/C) = kt, where C is AO7 concentration and t irradiation time (Figure 7) and are 3.2 h^−1^ and 1.0 h^−1^ for the experiments conducted with 10 mg L^−1^ and 25 mg L^−1^, respectively. The AO7 degradation rate decreased with the increase in dye concentration not simply because of the relative amount of dye molecules and available catalyst surface area, but due to the formation of higher concentrations of degradation products for the assay with 25 mg L^−1^, which will also adsorb on the catalyst surface, slowing the AO7 degradation mechanism.

For the AO7 initial concentration of 25 mg L^−1^, it is possible to see that the mentioned apparent rate constant was calculated for the first 4 h, followed by a visible decrease in the slope for the final hour of the assay. This is in accordance with the detection of several by-products during the photocatalytic assay, and their degradation and/or counter diffusion occurring in a way that, for some of the compounds, total degradation was also observed.

### 3.5. Toxicity

Both initial solutions were below the dye EC_50_ (*D. magna*) of 57.26 mg L^−1^. However, given the fact that some metabolites can be significantly more toxic than the parent compound, it is extremely important to monitor the toxicity of the final solutions to evaluate if the photocatalyst used is efficient enough to produce non-toxic final solutions.

For the ecotoxicity evaluation, acute toxicity (immobilization) assays with *D. magna* were performed for the solutions obtained after 2 and 6 h, for both initial dye concentrations. The EC_50_ was assessed by performing the acute immobilization test on a set of dilutions of each solution. The obtained result is expressed in concentration (%) of the initial solution, estimated to immobilize 50% of the *D. magna* within 48 h. The assay performed with 10 mg L^−1^ AO7, for both samples after 2 and 6 h, showed no toxicity to the tested organism. These results are in accordance with the detection of only small carboxylic acids at both 2 h (maleic acid) and 6 h (maleic, acetic, and formic acid), which are known to have very low toxicity (EC_50_ (*D. magna*) ≥ 48.81 mg L^−1^). However, for the assay with 25 mg L^−1^ AO7, after 2 h, an EC_50_ value of 61.21% was obtained. This could be explained by the presence of AN in a concentration of 0.87 mg L^−1^ in the 2 h sample, which is known to be a very toxic compound [38,39], with a 48 h EC_50_ (*D. magna*) value of 0.37 mg L^−1^. Nevertheless, as this metabolite suffered complete degradation until the end of the photocatalytic assay, the final solution (6 h) showed no toxicity to *D. magna*. These results show that the application of Sr_0.95_Bi_0.05_TiO_3_ as a photocatalyst in the degradation of the AO7 dye with visible light is efficient enough to also degrade the toxic metabolites successfully, producing final solutions non-toxic to *D. magna*.

The Sr_0.95_Bi_0.05_TiO_3_ oxide showed significant photocatalytic activity for the degradation of the AO7 dye under visible light, when compared with other metal oxide catalysts (Table 3). 

## 4. Conclusions

Bi-doped SrTiO_3_ perovskite (Sr_1−x_Bi_x_TiO_3_, x = 0, 0.03, 0.05, 0.07 and 0.1) powders were synthesized and applied as photocatalysts in the degradation of AO7 solutions under visible light. All doped samples exhibited a significant improvement in their photocatalytic activity when compared with the undoped SrTiO_3_. This improvement can be associated with the presence of a secondary phase, Bi_4_Ti_3_O_12_, and a possible synergistic effect between both phases. The possible formation of a heterostructure was also theorized, but further studies need to be performed. The best results were obtained with the Sr_0.95_Bi_0.05_TiO_3_ perovskite (0.2 g L^−1^), achieving complete AO7 degradation after 2 h and 3 h, for initial dye concentration of 10 mg L^−1^ and 25 mg L^−1^, respectively, at natural pH and under visible light. The ecotoxicity of the solutions was evaluated through acute toxicity assays with *D. magna*, and the solutions were found to be non-toxic after 2 h and 6 h for the initial dye concentrations of 10 mg L^−1^ and 25 mg L^−1^, respectively.

## Figures and Tables

**Table 1:** Cell parameters, unit volume, crystallite size, and bandgap energies for the Sr_1−x_B_ix_TiO_3_ perovskites.

x	a (nm)	V (nm^3^)	Crystallite Size (nm)	E_g_ (eV)
0	0.39147	0.05999	77.95	3.43
0.03	0.39099	0.05977	59.63	3.66
0.05	0.39187	0.06018	65.87	3.65
0.07	0.39097	0.05976	62.49	3.65
0.1	0.39110	0.05982	53.66	3.66

**Table 2:** TOC removals (%) for the photocatalytic degradation of AO7 with Sr_0.95_Bi_0.05_TiO_3_ perovskite (0.2 g L^−1^) under visible light.

AO7 Initial Concentration (mg L^−1^)	TOC Removal (%)	
	2 h	6 h
10	11.6	42.3
25	14.4	19.4

**Table 3:** Comparing the photocatalytic activity of different photocatalysts on the degradation of aqueous solutions of AO7 under visible light radiation.

Photocatalyst	Catalyst Dose (g L^−1^)	C_i_ AO7 (mg L^−1^)	t (min)	Degradation Efficiency (%)	Ref.
7% Co doped CeO NPs	1.0	15	180	95.4	[52]
NiO NPs	1.0	10	160	90.2	[53]
AuPt/Bi_2_O_3_	0.5	5	60	~98	[54]
Fe(2.5)-TiO_2_	3	10	180	~90	[55]
BaFeO_3_	0.5	5	360	97	[56]
Ni/Sr_0.9_Bi_0.1_TiO_3_	NA	25	420	83	[41]
Sr_0.95_Bi_0.05_TiO_3_	0.2	10	180	98.9	[This work]

## Data Availability

Not applicable.

## Supplementary Materials

The following supporting information can be downloaded at: https://www.mdpi.com/article/10.3390/ma15072465/s1, Figure S1: Emission spectrum of the 300 W Ultra-Vitalux lamp, Figure S2: Relative Abs_484nm_ decay for the photocatalytic degradation of AO7, with Bi_4_Ti_3_O_12_, under visible light (C_i_ AO7 = 10 mg L^−1^, catalyst dose = 0.2 g L^−1^, natural pH), Figure S3: XRD patterns of Sr_0.95_Bi_0.05_TiO_3_ powder samples (a) before and (b) after application as photocatalysts, Figure S4: SEM micrographies of the Sr_0.95_Bi_0.05_TiO_3_ (a) before and (b) after application as photocatalyst, Figure S5: Sr_0.95_Bi_0.05_TiO_3_ band gap energy determination (a) before and (b) after application as photocatalyst.
