# Supplementary material for: Visible-Light-Driven AO7 Photocatalytic Degradation and Toxicity Removal at Bi-Doped SrTiO3
Source: Materials (Basel). 2022 Mar 27;15(7):2465. doi: 10.3390/ma15072465 (PMC8999963; doi:10.3390/ma15072465)
Supplement: Supplementary file 1 [file materials-15-02465-s001.zip › materials-1636869-supplementary.pdf]

Visible-Light-Driven AO7 Photocatalytic Degradation and Toxicity Removal at Bi-Doped $SrTiO_3$

Maria João Nunes*, Ana Lopes, Maria José Pacheco and Lurdes Ciríaco

Fiber Materials and Environmental Technologies (FibEnTech-UBI), Universidade da Beira Interior, R. Marquês de D'Ávila e Bolama, 6200-001 Covilhã, Portugal

*Correspondence: maria.nunes@ubi.pt

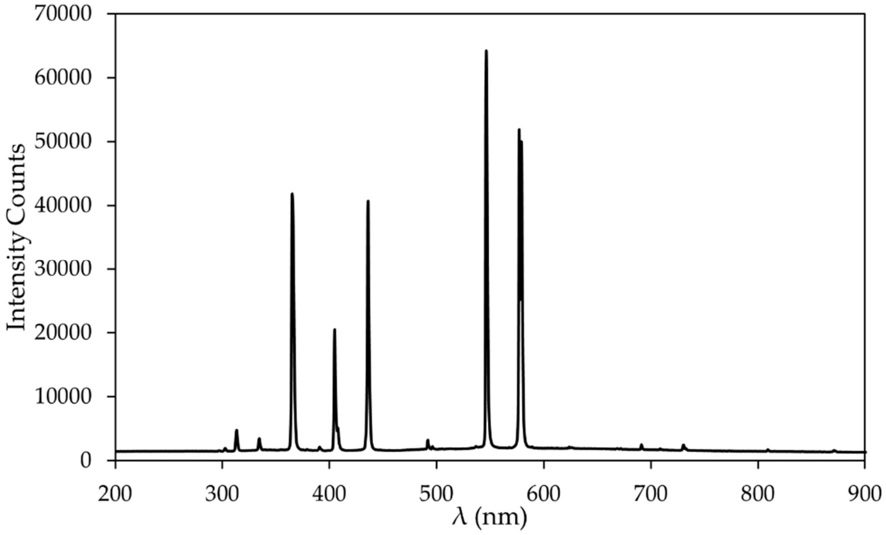

Figure S1. Emission spectrum of the 300 W Ultra-Vitalux lamp

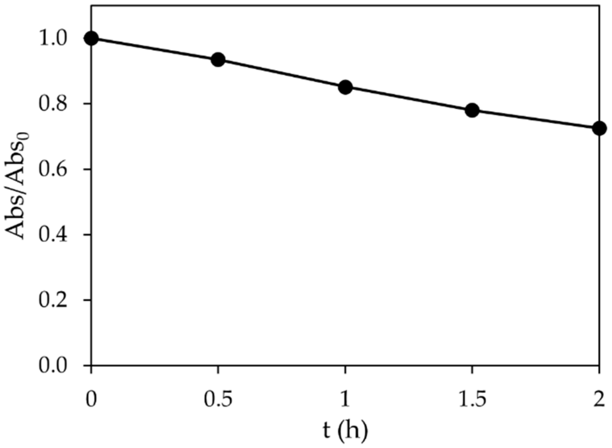

Figure S2. Relative Abs_{484nm} decay for the photocatalytic degradation of AO7, with $Bi_4Ti_3O_{12}$, under visible light (C_i AO7 = 10 mg L^{-1}, catalyst dose = 0.2 g L^{-1}, natural pH)

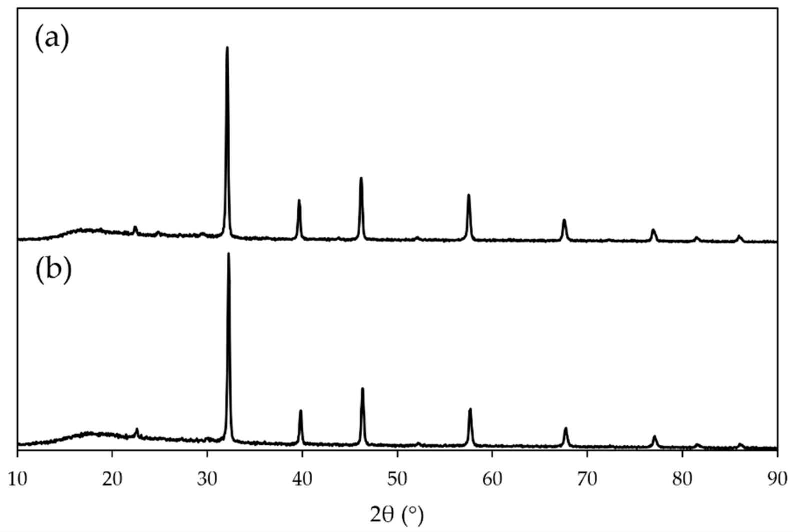

Figure S3. XRD patterns of $Sr_{0.95}Bi_{0.05}TiO_3$ powder samples (**a**) before and (**b**) after application as photocatalyst

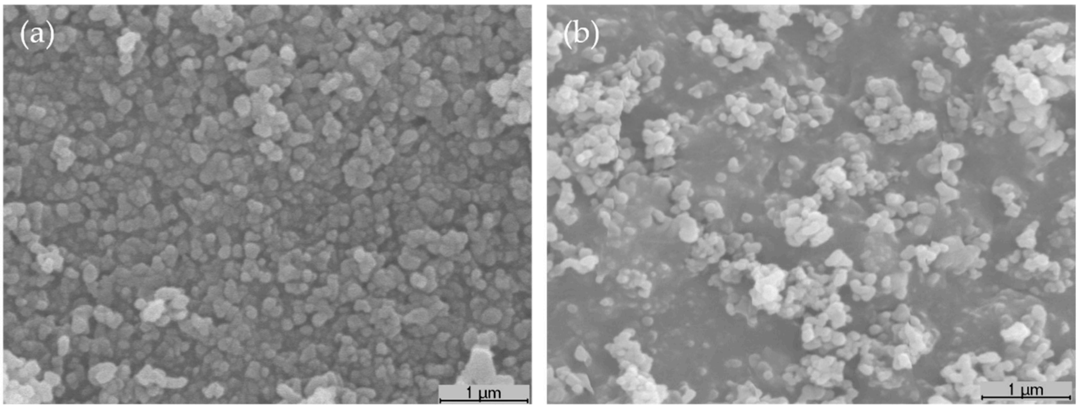

Figure S4. SEM micrographies of the $Sr_{0.95}Bi_{0.05}TiO_3$ (**a**) before and (**b**) after application as photocatalyst

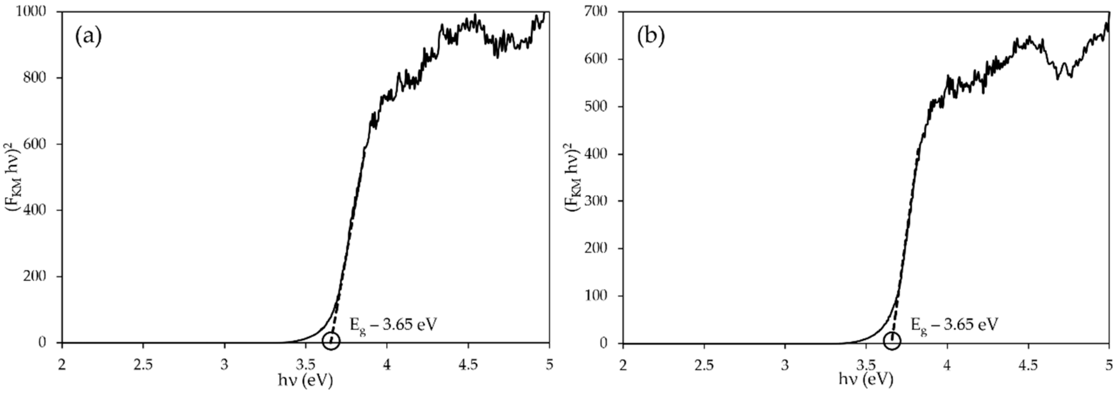

Figure S5. $Sr_{0.95}Bi_{0.05}TiO_3$ band gap energy determination (**a**) before and (**b**) after application as photocatalyst
